# Environmental factors associated with physical activity in childcare centers

**DOI:** 10.1186/s12966-015-0198-0

**Published:** 2015-03-29

**Authors:** Kathryn E Henderson, Gabrielle M Grode, Meghan L O’Connell, Marlene B Schwartz

**Affiliations:** Rudd Center for Food Policy and Obesity Yale University (at time of study), 309 Edwards Street, New Haven, CT 06511 USA

**Keywords:** Physical activity, Childcare, Environment

## Abstract

**Background:**

Child care centers influence physical activity levels among children, yet little is known about the specific aspects of the environment that support generous amounts of activity. The purpose of this study was to examine the practices, and environmental aspects of the child care center that are associated with children’s moderate and vigorous physical activity.

**Methods:**

Thirty-five child care centers serving 389 3 to 5 year old children were assessed for: 1) environmental characteristics of the center; and 2) staff practices related to child physical activity. Children’s physical activity was measured using accelerometers over a single day in child care.

**Results:**

Fourteen percent (an average of 9 minutes per waking hour) were spent in moderate to vigorous physical activity (MVPA). The strongest environmental predictors of MVPA were: time spent in outdoor play, suitability of indoor play space, and teacher encouragement of (but not participation in) indoor play.

**Conclusions:**

In order to reach the U.S. recommended 120 minutes of physical activity per day, significant changes will need to occur in the child care setting, including increased time outdoors and more opportunities for indoor physical activity.

## Background

The physical activity levels of children are influenced by environmental factors such as access to public recreation space and infrastructure, access to sidewalks, neighborhood crime and area deprivation [1-3]. Given the importance of establishing a pattern of regular physical activity early in life [4,5], understanding environmental factors that affect the activity levels of young children is essential.

Health organizations and government bodies around the world recognize the importance of physical activity in early childhood; however, many countries do not make specific recommendations about the amounts and type of physical activity necessary for children under the age of five. Among those that do, several recommend that children in this age group accumulate at least three hours of physical activity (any level) each day [6-8]. The Institute of Medicine in the U.S. similarly recommends young children obtain 15 minutes or more of physical activity each waking hour [9]. However, at the time of this writing, the U.S. National Association for Sport and Physical Education’s (NASPE) *Active Start* guidelines [10] state that children should acquire at least 60 minutes (up to several hours) of unstructured activity each day and at least 60 minutes of structured activity. U.S. National survey data on preschool aged children are not available; however, the U.S. National Survey of Children’s Health found that in 2011–2012, only 28% of U.S. children ages 6–17 participated in daily vigorous physical activity [11].

With approximately 60% of three to five year olds attending U.S. childcare centers [12], investigation of the characteristics that promote or hinder children’s physical activity in this setting is warranted. Prior research indicates that childcare centers play a major role in children’s physical activity. After controlling for individual-level factors, the childcare center can account for 14 to 47 percent of the variance in children’s physical activity [13-16]. For example, Pate and colleagues found that when controlling for children’s gender, age, race, ethnicity, BMI, and parent education, the preschool attended explained an additional 43.3% of the variance in moderate and vigorous activity [16]. Less is known about the specific aspects of the preschool environment that account for these differences [5,17]. In a review of the child care environment and children’s physical activity, Bower, et al. [17] identified factors that contributed to an environment supportive of physical activity: active opportunities (the combination of time allowed for outdoor play, structured activity, etc.), presence of portable playground equipment and physical activity training and education for children, staff and/or parents. Unexpectedly, this study also found that electronic media use was associated with higher levels of physical activity, while research by Dowda, et al. [18] found the opposite: lower media use was associated with higher levels of activity. Dowda and colleagues [18] also found that more portable equipment was associated with higher levels of activity, along with larger playgrounds. This body of research is small—more study is needed to identify features of the child care setting that promote physical activity in order to capitalize on childcare centers’ unique capacity to promote adequate engagement in physical activity among children.

The purpose of this study was to examine the practices and physical aspects of the childcare center environment that are associated with increased levels of children’s moderate and vigorous physical activity and compare children’s rates of physical activity to U.S. national recommendations. This research differs from previous studies, as it includes a large sample of centers and focuses on children from low-income, ethnically diverse populations. In this study, the childcare environment was audited, and children from 35 childcare centers wore accelerometers to objectively measure their activity levels throughout an entire preschool day. Findings from this study may inform state and federal policies, as well as policies of childcare accrediting agencies, that are designed both to improve children’s health and to enhance the overall quality of childcare centers.

## Methods

### Sample

This study was part of a larger project designed to document policies and practices in child care centers that participated in the U.S. Child and Adult Care Food Program (CACFP) and that served preschool-aged children (though not necessarily exclusively). Thus, some study criteria were dictated by the larger project goals. The CACFP supports food service in US-based non-profit child care centers, outside-school-hours care centers, Head Start programs, and for-profit early child education centers that enroll at least 25% of children from low-income families.

A pool of 221 licensed full and partial-day Connecticut childcare centers were identified that met the following inclusion criteria: 1) served at least 13 3–5 year olds; 2) participated in the Child and Adult Care Food Program; and 3) were not in-home facilities. From this pool, 40 centers were selected in a random stratified sample to ensure adequate representation of low-income communities. Three to five year old children attending these childcare centers were individually enrolled in the study; infants, toddlers and school-aged children enrolled in the centers were not included in the study.

### Procedure

All methods were approved by the Yale University Institutional Review Board. Children were enrolled in the study via parental consent.

### Environmental audit

Two domains of the physical activity environment were assessed through an environmental audit: 1) physical characteristics, which included items such as the size of the outdoor play space and the suitability of the indoor play space for a variety of activities; and 2) practices, which included items such as staff participation in outdoor play and staff provision of structured activities. Data on the physical activity environment were collected primarily through direct observation. The development of the audit is described in Henderson et al. [19] and the observation tools are included in the appendix. Researchers received several hours of training and practice using the audit. Multiple researchers were present at each site visit, and each researcher provided a rating on all audit items. Disagreements among researchers were resolved following the end of each visit.

Child-level variables were assessed through direct observation (i.e. gender), on-site measurement (i.e. height), and through information collected from parent consent forms (i.e. date of birth). Other variables, such as median household income of the census block where centers were located, were collected from online sources.

A number of variables were collected as part of the audit, but not included in analyses due to limited variability across sites. These included physical activity training for teachers, outdoor temperature, restriction of physical activity for punishment, teaching of physical education lessons, presence of video system/VCR/television, and quality of outdoor running space.

### Accelerometry

Actigraph GT1M accelerometers were used to assess children’s physical activity. Accelerometers are the most popular objective measurement device [20] and have been validated for use with the preschool population [21-23]. The Actigraph GT1M is small (1.5in x 1.44in x 0.70in), lightweight (27 grams) and unobtrusive to wear.

Research assistants received a two hour training on the use of accelerometers (how they should be fastened, etc.) and how to use the observation tool. The protocol was piloted in one center before beginning data collection and minor revisions were made. Two research assistants visited each preschool to conduct the environmental audit and the accelerometer portion of the study. They arrived at the beginning of the day, placed an elastic belt with an accelerometer on the child, which was worn over the right hip, and recorded the start and end of each child’s individual wear time. The research assistant was present for the full day to ensure that accelerometers were worn correctly, to record the length of various class activities, and to audit the environment. At the end of the day the research assistants compared and discussed the environmental assessments and resolved any discrepancies. The observations lasted an average of 5.13 hours (±1.52 hours). In data cleaning, the portion of accelerometer data corresponding to each center’s naptime was removed in order to focus analyses on waking hours. Therefore, on average, accelerometers were worn for 3.33 hours (±0.7 hours). Accelerometers were numbered and linked to each child by name. The data were downloaded daily to a computer.

Accelerometers were programmed to collect physical activity data in five second sampling intervals in order to capture young children’s short bursts of movement [24,25]. Cut-points for activity levels derived by Evenson and colleagues were applied to the accelerometer data [26]. Evenson’s cut-points were tested with 5–8 year olds and fall in the middle of those used in three other calibration studies conducted with 3–16 year olds [27-29].

Each interval was categorized as sedentary (≤8 counts per 5-sec interval), light (9–191 counts per 5-second interval), moderate (192–334 counts per 5-second interval) or vigorous (335–1400 counts per 5-second interval). Counts beyond 1400 per 5-sec interval were considered outliers and were excluded. Sixty minutes (720 5-second intervals) of consecutive zeros were considered non-wear time.

### Body mass index

Children’s height was measured to the nearest 0.25 inch using Seca 214 portable stadiometers. Their weight was measured to the nearest 0.1 lb with Seca Clara 803 digital scales. BMI percentile was calculated using the Centers for Disease Control and Prevention (CDC) Children’s BMI Tool for Schools [30]. Using CDC’s criteria for categorizing weight status, children between the 85^th^ and 94^th^ percentile were considered overweight, while children in the 95^th^ percentile and above were considered obese.

### Statistical analyses

Accelerometer data were processed with MeterPlus software [31] and analyzed using SPSS [32]. The dependent variable was the proportion of counts spent in moderate and vigorous activity (MVPA) compared to total wear time. The majority of the environmental predictor variables (center-level) were categorical (i.e. yes/no). However, several variables were originally continuous and subsequently categorized in analysis, such as fixed equipment. The decision on how to categorize these variables primarily came from a previous study conducted by Dowda and colleagues [18], in which variables were split by quartiles or the median. Duration of outdoor play was categorized according to whether the center provided 60 minutes or more of outdoor play, which follows Dowda’s scheme and is also a commonly used benchmark for adequate amounts of physical activity [33].

Linear mixed models were used in order to account for children nested in classrooms and to simultaneously study child-level and center-level predictors. For each model, the preschool site was entered as a random effect; all other variables were entered as fixed effects. The method of estimation was restricted maximum likelihood. The significance of independent variables was tested, controlling for significant demographic factors, which included gender, age, and BMI percentile.

## Results

Thirty-five centers out of the original 40 were included in analyses. Two centers declined to participate in the study, and three centers were excluded because either a site visit could not be scheduled during the study period or normally scheduled physical activity sessions did not occur during the site visit. For a few of the site-level variables, one site was missing data; for these analyses, the overall N is reduced by 1 and, at the child level, by the number of children within said site. Further, we were able to observe indoor play at only 24 sites, so those analyses have reduced overall N as well. Sample sizes for each analysis are noted in Table 1, where results are reported. One-half were Head Start sites, and 57% were accredited by the National Association for the Education of Young Children. Twenty-seven percent of the centers were half day programs and 73% of the centers were full day.

**Table 1:** **Adjusted mean percent of moderate and vigorous physical activity (as a function of total accelerometer wear time) by center-level predictor variables in linear mixed models**

**Predictor Variable**	**Description**	**N**	**Adjusted Mean Percent of MVPA (SE)**
Physical Characteristics			
Suitability of indoor playspace	Quiet play	6	12.0 (0.9)*
	Limited movement	23	13.6 (0.5)
	All activities	5	14.9 (1.0)
	(referent category)		
Posters, books, pictures of PA in class	Yes	18	14.8 (0.7)^+^
	No	17	13.0 (0.7)
Classroom area (upper quartile split)	<1248 ft^2^	26	13.5 (0.6)
	≥1248 ft^2^	8	15.0 (1.0)
Playground area (upper quartile split)	<5436 ft^2^	25	13.7 (0.6)
	≥5436 ft^2^	10	14.3 (0.9)
Fixed equipment (median split)	<10	15	13.3 (0.7)
	≥10	19	14.5 (0.7)
Portable equipment (median split)	<21	16	14.5 (0.7)
	≥21	18	13.5 (0.7)
Sedentary equipment (median split)	<9	18	14.4 (0.7)
	≥9	16	13.5 (0.7)
Drinking water available outdoors	Yes	9	14.7 (0.6)
	No	26	13.6 (1.0)
Practices			
Duration of outdoor play	<60 minutes	27	13.4 (0.5)*
	≥60 minutes	8	15.8 (1.0)
Num. of children on playground (median split)	<16	16	14.4 (0.7)
	≥16	19	13.4 (0.7)
Teacher-led outdoor PA	Yes	22	13.3 (0.8)
	No	13	14.2 (0.6)
Staff participation in indoor play	Yes	17	13.3 (0.7)*
	No	7	16.2 (1.1)
Staff participation in outdoor play	Yes	15	13.3 (0.7)
	No	20	14.3 (0.6)
Staff encouragement of PA in indoor play	Yes	12	15.3 (0.8)*
	No	12	12.9 (0.8)
Staff encouragement of PA in outdoor play	Yes	12	13.5 (0.8)
	No	23	14.1 (0.6)
Computer availability (to children) per observation day	≤15 minutes	22	14.5 (0.6)
	(including no		
	computer)		
	>15 minutes	13	12.9 (0.8)
PA curriculum	Yes	13	13.3 (0.8)
	No	22	14.2 (0.6)
Other			
Median household income of preschool block group^1^	Continuous (dollars)		Coeff = .003651 (.001515)*
NAEYC^2^	Yes	20	13.8 (0.6))
	No	15	14.0 (0.8)
Head Start^2^	Yes	18	13.4 (0.7)
	No	17	14.4 (0.7)

Estimated marginal means, controlling for gender, age, and BMI percentile. Variables collected during on-site observation unless otherwise noted.

^1^ U.S. Census Bureau.

^2^ Interview.

* p < 0.05.

^+^ borderline significant (p = 0.064).

A total of 447 children participated in the study, representing participation of over 90% of eligible children at each center. Non-participation was due largely to parents or guardians not returning the required paperwork in time for the study. Fifty-eight children were excluded from analyses due to missing data. Missing data were most commonly due to children who removed the accelerometers prior to the end of the observation period or damaged them during wear, or the (unexplained) malfunctioning of accelerometers simply resulting in failure to record data throughout the wear period. Additionally, we were missing demographic data for 15 children, thus, those children were excluded from analyses. Of the 389 available for analyses, 50% of children were male, 50% were Hispanic, 49% were white, 34% were black, and their mean age in months was 56.4 (±7.9) (4.7 years). The mean BMI percentile was 60.2 (±31.5); 31% of children were overweight or obese.

**Figure 1:**
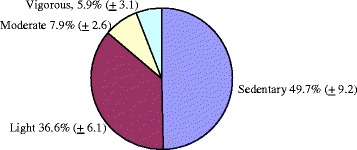
**Average percent of observed time spent in physical activity levels, excluding naptime.**

Childcare centers, on average, allotted 23.0% (±10.6) of their schedule to indoor or outdoor physical activities; 20.6% (±9.6) to free play activities in which children choose classroom activities in which to participate; 28.9% (±11.3%) to sedentary activities, such as meal time, circle/story time, or structured lessons; and 27.6% (±17.8%) to naptime. Excluding the nap period, children spent an average of 14% of observed time in MVPA (see Figure 1). This translates to nine minutes per hour of MVPA during non-nap time. Across the average day of observation, children accrued 27 minutes of MVPA.

The results of the linear mixed models for the child-level characteristics (considered control variables in subsequent models) indicate that male children engaged in significantly more MVPA than female children (15.1% of time in MVPA vs. 12.7%, p < .001). Further, older preschoolers were significantly more active, p < .001) as were heavier preschoolers (defined by BMI percentile, p < .01).

Table 1 shows the results of linear mixed model analyses for center- level characteristics, controlling for the child-level characteristics of BMI percentile, gender and age (these findings are reported above). Given the categorical nature of the predictors, adjusted means and their accompanying standard errors are presented. Model coefficients for the non-referent group category may be derived as the difference between estimated marginal means. Several significant center characteristics emerged as important predictors of physical activity. Children had significantly higher levels of MVPA in centers where outdoor play was 60 minutes or longer; indoor classroom space was suitable for all types of activities; staff encouraged children to increase their physical activity during indoor play; and the neighborhood in which the preschool was located had a higher median household income. There was also a positive trend for the presence of posters, books, and pictures featuring physical activity. Centers in which staff participated in indoor play had lower levels of MVPA.

## Discussion

The purpose of this study was to determine the factors associated with higher levels of MVPA among a large sample of ethnically diverse, low-income, U.S. preschoolers. This study extends previous research which showed that preschools account for a significant amount of the variance in children’s physical activity [13-16] and also builds upon more recent work which explores the specific characteristics of childcare centers that are related to increased levels of physical activity [17,18].

Only 14% of observed time was spent in MVPA, which is consistent with prior research [16,17]. This represents an average of 9 minutes per hour, or 27 minutes over the course of the preschool day. Because data were collected in the spring and early summer months and because site visits were purposely scheduled on non-rainy days in order to observe outdoor play, this amount of physical activity is likely an optimistic accounting of MVPA. During other times of the year, the amount of MVPA in which children engage is likely to be lower [34]. It also unlikely that children in full-day childcare are obtaining more MVPA after school [35,36], as this time is likely to be spent at home during dinner and bedtime. Therefore, it is unlikely that preschool children are obtaining the amounts of daily activity recommended by NASPE in the U.S. nor those recommended by health organizations abroad [6-8,10]. For this reason, it is recommended that childcare centers attempt to increase opportunities for MVPA during the day.

Consistent with previous research, our study found that males were significantly more active than females [4,13-16,37,38]. Age (older) and BMI (heavier) were also significant predictors of increased MVPA. The BMI finding was surprising, because others have found no association between BMI and physical activity in preschoolers [13,14,16,38,39].

Also consistent with other studies, the duration of outdoor play had a significant positive association with children’s MVPA [4,17,38-40]. In fact, some researchers posit that because of this strong association, subjective reporting of time spent in outdoor play can even be used as a proxy measure for actual physical activity [41]. Considering the lack of physical activity regulations specific to child-care centers, mandating explicit amounts of physical activity could have a considerable impact on children’s activity levels. Indeed, the New York City Health department has a policy that all full day programs must provide 60 minutes of physical activity per day [42], and three U.S. states, Alaska, Delaware, and Massachusetts, have physical activity policies that specify a daily number physical activity minutes [43]. However, while cross sectional studies strongly suggest that children who spend more time playing outdoors are more physically active than those who spend less time outdoors, experimental studies are needed to confirm that increasing outdoor play time is an effective strategy to increase physical activity in child care. Only one randomized controlled trial to date has tested this strategy. No significant differences in time spent in MVPA during the entire day or the child care day were found between children assigned to usual amounts of outdoor play and those assigned two additional 30 minute periods of outdoor play during the child care day [44]. Further research is needed.

A literature review of seven accelerometer-based studies that took place in the U.S. (four studies), Scotland (two studies) and Belgium (one study), included 63 different centers and about 1,000 children, found that no centers provided 60 minutes of MVPA [45]. Increasing physical activity opportunities in child-care centers would not cause a financial strain. The challenge for centers would be in scheduling additional physical activities while maintaining the integrity of school readiness activities [46]. In scheduling more active play, centers should consider offering shorter and more frequent opportunities for active play, as opposed to one long play period, as young children will not be able to maintain moderate/vigorous activity for extended bouts [47].

Given the potential importance of outdoor play in promoting MVPA, an emphasis should be placed on ensuring that children are adequately prepared to be outdoors, which includes proper sun protection and appropriate outerwear and shoes. Childcare centers may skip outdoor play for the entire day if a single child is not dressed appropriately [48,49]. Centers will have to work with parents to ensure that they dress their children appropriately and may also consider creating a communal box of clothing and sun protection items. In the context of limited resources, putting funding toward this endeavor would be worthwhile.

Several aspects of the indoor environment were associated with MVPA, which indicates that the indoor area should not be overlooked as an important site for physical activity. The suitability of the classroom for all activities, staff encouragements to increase physical activity during indoor play, and the presence of physical activity books, posters, and pictures were all associated with higher levels of MVPA. The presence of physical activity materials may be indicative of a center culture that promotes activity. All three of these factors represent no- or low-cost improvements that can be easily implemented in child-care centers. For example, furniture can be moved to allow for a small movement corner. These factors, however, have not been assessed extensively, and therefore it is not yet known if they are reliably associated with MVPA. Future research should include exploration of these aspects of the indoor environment, especially in light of the low costs associated with altering them.

Unlike staff encouragement of physical activity indoors, encouragement outdoors was not a significant predictor of MVPA, which could indicate that children do not need prompting to be active when outdoors. In contrast, a small pilot study with five preschool children showed that teacher-encouraged activities increased MVPA during outdoor play [50]. More research is needed to further assess the impact of staff encouragement on children’s physical activity levels.

The final variable significantly and positively associated with MVPA was the median household income of the neighborhood (defined as census block group) in which the center was located. All of the participating childcare centers were located in low income communities. Even within this restricted sample, the higher income neighborhoods were linked to higher MVPA. This finding may be related to issues of neighborhood safety and quality, which could impact centers’ operations in a variety of ways. In a literature review of environmental attributes that influence children’s physical activity, Davison and colleagues reported that objective measures of crime rates and area deprivation were significantly, negatively associated with children’s physical activity [3]. Childcare centers located in lower income neighborhoods may need to utilize their indoor play spaces more extensively and secure safe outdoor spaces for play. It is important to note that this finding in our sample is correlational – thus, greater MVPA in higher-income neighborhoods may be produced by an unmeasured variable.

One environmental factor, staff participation in indoor play, was significantly and negatively associated with MVPA. This finding was surprising and warrants additional exploration. Observational learning via modeling is considered an important element in learning and health theories [51]. Based on this premise, it was expected that if teachers also participate in physical activity, children’s activity levels would increase. In conversations with childcare teachers, they mentioned that a major barrier to children’s physical activity was teachers who do not participate in physical activities with the children. A possible explanation for this finding is that in indoor environments, which are limited in space, adults decrease the activity level by reducing space available to children, whereas outdoors, space is generally adequate to accommodate full movement of all children and adults. An alternative explanation may be that there are more safety concerns indoors to which adults respond when they participate in the activity, which might lower activity levels. Finally, teachers may tend to initiate their own participation more when children are less active – that is, increased teacher participation is an *effect* rather than a cause of lower MVPA in children. Further research is need to explore this association.

Conversely, staff participation in outdoor play was not significantly associated, positively or negatively, with children’s MVPA. Structured physical activity, in which teachers led activities indoors or outdoors, was also not significantly associated with increased MVPA. Intervention studies that have assessed the impact of increased structured physical activity on total daily physical activity have had mixed results [52]. Additionally, increased teacher-led physical activity may compromise the amount of time children are able to engage in self-directed play, which is important for child development [52].

Many of the environmental factors measured did not have significant associations with MVPA in this sample, such as outdoor play area size, types of play equipment, electronic media, and physical activity policies. Some of these factors, like electronic media, have inconsistent results across studies [38], while others, such as types of play equipment [17,18,53-55] and open space [56], have a small supportive evidence base. Discrepancies in findings between this study and other studies may be attributed to the different ways in which variables were measured and to the sample population, which, in our study, included primarily low-income childcare centers and ethnically diverse preschoolers. It is also important to acknowledge that this field is relatively new and that as researchers continue to analyze which preschool environmental factors are associated with MVPA, evidence will build.

Limitations of this study include its cross-sectional design and the observation of physical activity on only one day, with an average of only about three hours per child. This study is also limited in the way that all studies relying on accelerometry are limited: currently there are no universally agreed upon cut-off points for physical activity levels among young children [57]. Additional study is needed to determine the most appropriate cut-off points for this population. Bornstein, et al. [58] recommend that a conversion system be used to allow comparison of results across studies where varying cut points were used, and have developed prediction equations to allow for direct comparison between studies employing different physical activity level cut-off points [59]. Further research should make use of this tool to compare data across studies. Finally, researchers were present and observing for the better part of the day. It is possible that this could have influenced child behavior, or more likely, child care center staff practices that may have influenced child physical activity levels.

## Conclusion

With guidance on structuring the environment to promote activity, childcare settings are optimally positioned to improve the health and well-being of young children by increasing their physical activity levels. Experimental research is needed to identify effective strategies to use in this setting. In the absence of these type of data, we recommend the following based on results of the current observational study:

- Full-day centers provide at least 60 minutes/partial day center provide at least 30 minutes of outdoor play, offered throughout the day
- Centers create space, even small areas, suitable for active play in their indoor environments
- Centers include positive messages about physical activity in their classrooms and communal areas
- Centers train teachers to verbally encourage safe physical activity, particularly indoors

These recommendations, some of which have also been suggested by other researchers [60], represent strategies that are generally inexpensive and easy to implement. Childcare accrediting agencies, developmental experts, and public health advocates must work together to institute changes to the childcare environment that promote physical activity without compromising the integrity of other curriculum foci.
